# Rapid quantification and PK-PD modeling of rocuronium bromide in beagles using portable mass spectrometer

**DOI:** 10.3389/fvets.2025.1543086

**Published:** 2025-03-04

**Authors:** Xiaoxiao Li, Pan Chang, Xing Liu, Deying Gong, Wensheng Zhang

**Affiliations:** ^1^Department of Anesthesiology, West China Hospital, Sichuan University, Chengdu, China; ^2^Laboratory of Anesthesia and Critical Care Medicine, National-Local Joint Engineering Research Centre of Translational Medicine of Anesthesiology, West China Hospital, Sichuan University, Chengdu, China

**Keywords:** rapid analysis, rocuronium bromide, mass spectrometer, HPLC-MS, beagle

## Abstract

Monitoring rocuronium bromide (Rocur) concentrations is crucial for assessing muscle relaxation in clinical anesthesia. However, no suitable instruments are currently available. This study explores the application of a portable mass spectrometer (MS) for the rapid detection of Rocur concentrations in whole blood from beagles, aiming to support the development of individualized pharmacokinetic-pharmacodynamic (PK-PD) models. Four beagles (1–1.5 years old, 8–12 kg) received a single intravenous dose of Rocur (3 ED₅₀, 0.748 mg/kg). Neuromuscular monitoring was conducted using the train of four (TOF) ratio. Blood samples (0.1 mL) were collected at predetermined intervals and during recovery, with TOF ratios recorded at corresponding time points. Rocur concentrations in whole blood (C_b-Rocur_) were quantified using both the Cell portable MS and high-performance liquid chromatography with mass spectrometry (HPLC-MS) for consistency assessment. Additionally, a PK-PD model was developed based on C_b-Rocur_ measurements obtained from the Cell portable MS. A strong linear relationship was observed for Cell portable MS measurements within the range of 50–10,000 ng/mL (*y* = 1108.32 * *x* + 14873.99, *R*^2^ = 0.993), with a limit of quantification (LOQ) of 50 ng/mL and a limit of detection (LOD) of 10 ng/mL. A strong linear correlation was found between the two techniques (*y* = 1.07 * *x* + 30.08, *p* < 0.0001, *R*^2^ = 0.8948), with a relative standard deviation <15% for all concentrations. The C_max_ values were 4.52 ± 1.16 μg/mL (Cell portable MS) and 4.89 ± 0.52 μg/mL (HPLC-MS), respectively. As C_b-Rocur_ decreased, the TOF ratio gradually recovered, with an IC_50_ of 0.25 ± 0.05 μg/mL. This study successfully applied the Cell portable MS for rapid quantitative Rocur analysis in whole blood, demonstrating high consistency with HPLC-MS. The findings also revealed the good correlation between the PK-PD properties of Rocur and TOF effects.

## 1 Introduction

Rocuronium bromide (Rocur), a widely used non-depolarizing muscle relaxant in clinical anesthesia, offers benefits such as rapid onset of action, minimal metabolic byproducts, and negligible cardiovascular side effects (1, 2). Rocur induces muscle relaxation by competitively binding to N-type acetylcholine receptors at motor nerve synapses, inhibiting the action of acetylcholine (3, 4). However, substantial interindividual variability in the ADME of Rocur influences pharmacokinetic parameters, including the time to maximum concentration (T_max_), maximum concentration (C_max_), clearance, volume of distribution, elimination half-life, consequently resulting in unpredictable action duration and dose–response relationships (5). This variability can lead to either underdosing or overdosing during surgery. Underdosing may cause early recovery of muscle relaxation and abdominal tension during laparoscopic procedures, raising the risk of postoperative umbilical hernia. Conversely, overdosing may cause residual neuromuscular blockade (RNMB) after extubation, potentially resulting in incomplete respiratory recovery or even life-threatening complications (6, 7).

Due to technology limitations in rapid drug concentration analysis, clinicians have traditionally used acceleromyography (AMG)-based neuromuscular stimulation to roughly estimate RNMB (8), which suffers from data inconsistencies, especially in awake postoperative patients, where involuntary thumb movements can interfere with measurements and result in clinical misjudgments, potentially leading to premature or delayed extubation (9, 10). Thus, the capability to rapidly and accurately monitor Rocur concentrations in whole blood (C_b-Rocur_) is vital for precise individualized dosing and ensuring the safety of anesthetized patients.

The Cell portable mass spectrometer (MS), based on in-situ ionization technology and ion trap MS, offers notable advantages such as compact size and strong portability. Previous studies have demonstrated its effectiveness in the rapid identification of specific compounds in traditional Chinese medicine (11). This study aims to investigate the application of the Cell portable MS for the rapid detection of C_b-Rocur_ and the development of individualized pharmacokinetic-pharmacodynamic (PK-PD) models. We hypothesize that the Cell portable MS has significant potential for rapid monitoring of intravenous anesthetic concentrations for guiding personalized anesthesia administration.

## 2 Materials and methods

### 2.1 Ethics approval

This study involved 4 beagles (purchased from Chengdu Dossy Technology Co., Ltd.), aged 1 to 1.5 years and weighing 8 to 12 kg, with no sex restrictions. Blood biochemical tests were conducted, and beagles with abnormal results were excluded. Ethical approval was obtained from the Animal Ethics Committee of West China Hospital, Sichuan University (20240930006), and all procedures complied with animal welfare guidelines.

### 2.2 Animals preparation

**Figure 1:**
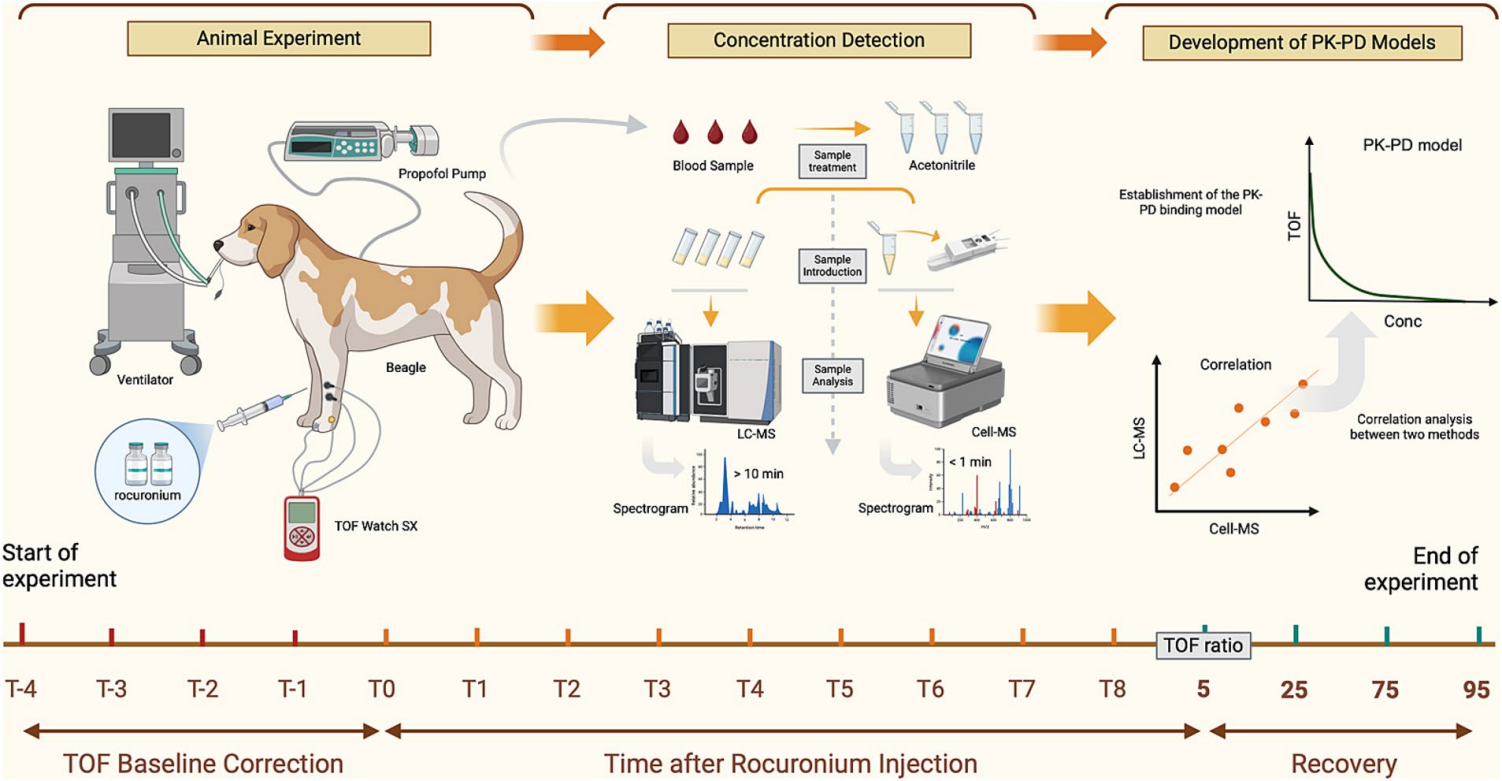
Workflow for the analysis of Rocur using the Cell portable MS. Compared to the LC–MS, Cell portable MS significantly reduces sample pretreatment and chromatographic separation time. T_−4_ to T_−1_ represents the calibration time for TOF watch; T_0_ to T_8_ refers to blood sample collection time points, including before drug administration (0 min) and at intervals from 0.5 min to 30 min (if applicable) after Rocur administration. TOF ratios of 5, 25, 75, and 95% indicate additional sample collection time points when the TOF ratio first increases to 5–25%, 25–75%, 75–95%, and above 95%, respectively.

All beagles were fasted for 12 h before the experiment, with free access to water, to minimize the effects of gastric content on Rocur absorption and metabolism. As shown in Figure 1, a 20 G intravenous catheter (Surflo Flash, Terumo, Japan) was inserted in the left forelimb vein for drug administration, and another 20 G catheter was inserted in the left hindlimb vein for blood sampling. Anesthesia was induced with intravenous propofol (3 mg/kg), with additional doses (0.5 mg/kg) titrated as needed to achieve the appropriate anesthetic depth based on the beagle’s response. Anesthesia was maintained with a continuous infusion of propofol (0.17 mg/kg/min). Once the righting reflex was lost, the beagles were positioned on a heated operating table, with body temperature maintained at 36.5 ± 0.5°C throughout the procedure. Vital signs including heart rate, electrocardiogram, blood pressure, and pulse oxygen saturation were monitored using the monitor (T8 View, Shenzhen Mindray, China). A 20 G intravenous catheter (Surflo Flash, Terumo, Japan) was inserted in the femoral artery for invasive blood pressure monitoring, with systolic blood pressure maintained within ±20% of the preoperative baseline. Tracheal intubation was then performed, and the beagles were connected to a ventilator (Boaray 700, Prunus, China) via a re-breathing circuit with the following settings: volume control ventilation mode, pure oxygen supply, a flow rate of 2 L/min, a respiratory rate of 20 breaths/min, and a tidal volume of 10 mL/kg. Due to the short experiment duration, the beagles did not receive additional fluid supplementation. Upon full recovery and the restoration of independent ambulation, the beagles were returned to the animal room. If residual neuromuscular blockade persisted, it was reversed with neostigmine (0.03 mg/kg) and atropine (0.03 mg/kg), with additional doses administered as needed.

### 2.3 Neuromuscular function monitoring

The neuromuscular function monitoring was conducted following the methodology described in previous studies (12). The beagles were positioned in right lateral recumbency, and two silver needle electrodes were inserted subcutaneously, approximately 2 cm apart, between the lateral femoral condyle and the proximal one-third of the fibula to stimulate the peroneal nerve. The stimulation needles were connected to an AMG monitor (TOF-Watch SX; MSD R&D (China), China). The AMG monitor’s acceleration sensor was affixed between the third and fourth dorsal toes using surgical tape. Skin surface temperature was measured by attaching the AMG monitor’s temperature sensor near the proximal tibialis anterior muscle.

Neuromuscular function was evaluated using the AMG monitor in train-of-four (TOF) stimulation mode with the following parameters: a pulse duration of 0.2 ms, a frequency of 2 Hz, and an interval of 15 s. TOF stimulation of the peroneal nerve was initiated 15 min before Rocur administration to minimize enhanced muscle contractions that could introduce measurement errors (13). The CAL2 function of the AMG monitor was then used for calibration. TOF ratios (TOFR) were continuously recorded with TOF-Watch SX (Version 2.5.INT, Organon, Ireland). If significant changes in TOFR occurred, a 15-gram weight was attached to the dorsal surface of the third and fourth toes. Calibration was considered complete if the TOFR variation was below 5% for 5 min. Following calibration, the control TOFR and the supramaximal stimulation current were recorded before drug administration.

### 2.4 Rocur administration

Following continuous propofol infusion, the beagles lost consciousness and maintained stable respiration, indicating an appropriate anesthetic plane. At this point, Rocur (Rocuronium Bromide Injection; Zhejiang Xianju, China) was administered intravenously. To simulate clinical dosing, a single intravenous dose of 3 ED₅₀ (ED₅₀ = 0.248 mg/kg, 3 ED₅₀ = 0.744 mg/kg) was given, based on data from a preliminary study involving 15 beagles conducted at this research center. Rocur was administered within 30 s at a constant rate of 1.5 mg/kg/min using a calibrated micro-infusion pump (BeneFusion n series, Shenzhen Mindray, China).

### 2.5 Blood sample collection and analysis

#### 2.5.1 Sampling time points and sample pre-treatment

Whole blood samples were collected at the following time points: before drug administration (0 min), 0.5, 1, 3, 5, 7, 10, 12, 15, 20, and 30 min (if applicable) after the drug administration. Additional samples were taken when the TOFR first increased to 5–25%, 25–75%, 75–95%, and above 95%. These TOFR intervals correspond to muscle relaxation stages described in *Miller’s Anesthesia*: TOFR <5% indicates a profound block, 5–25% a deep block, 25–75% a moderate or surgical block, and > 95% recovery from neuromuscular block. At each specified time point, 0.1 mL of blood was drawn from the left hindlimb and precisely measured with a 100 μL pipette. The sample was immediately transferred into an EP tube containing 400 μL of HPLC grade acetonitrile (≥99.9%, Biologic-Reagents, China) and thoroughly mixed for subsequent analysis.

#### 2.5.2 HPLC-MS detection

Quantitative analysis was conducted using a high-performance liquid chromatography with mass spectrometry system (HPLC-MS, Agilent 1260, Japan). LC Conditions: The chromatographic column was an Ultimate XB-C4 (3.0 mm × 100 mm, 3 μm, 300 Å, Welch Materials, USA). The mobile phases consisted of Phase A (0.1% formic acid aqueous solution) and Phase B (acetonitrile). The flow rate was maintained at 0.3 mL/min. Phase B was initially set at 25% and held for 2 min, then linearly increased to 60% from 2 to 4 min, and further increased to 90% at 4.1 min. The total runtime was 7 min, followed by a 2.5-min column equilibration. The column temperature was maintained at 30°C, and the injection volume was 1 μL. MS Conditions: An electrospray ionization source was employed in positive ion detection mode with multiple reaction monitoring. The drying gas temperature was 350°C, with a flow rate of 5 L/min. The nebulizer pressure was set at 45 psi, and the sheath gas temperature was 350°C with a flow rate of 11 L/min. The capillary voltage was set to 3,500 V. For Rocur, the ion transition was m/z 529.4 → 487.3, with a fragmentor voltage of 134 V and a collision energy of 32 V. For verapamil, the ion transition was m/z 455.3 → 165.2, with a fragmentor voltage of 150 V and a collision energy of 28 V.

#### 2.5.3 Cell portable MS detection

The Cell portable MS (C3001-sci, Suzhou Purspec Technology, China) is a tandem mass spectrometry-based analytical instrument that consists of an ion source, ion transmission system, linear ion trap, ion detection device, and control system. The ion source ionizes samples to generate ions, which are transmitted via the ion transmission system to the linear ion trap. Within the ion trap, ions are stored, selected, and fragmented before being sequentially transmitted by mass-to-charge ratio to the ion detection device. The entire process is managed by an integrated control system. This device measures 333 × 235 × 146 mm, weighing less than 8.5 kg and offering a mass range of 50–1,000 m/z, supports switching between positive and negative ion modes. Its sample introduction interface uses a discontinuous atmospheric pressure interface. It enables selective precursor ion fragmentation and multistage mass spectrometry analysis of substances, including isomers, providing results within 10 s.

Experimental Setup: MS scanning was conducted in positive ion mode. The precursor ion of Rocur was m/z 529.3 with an ionization energy of 1.3 V, and the fragment ion was m/z 487.3. A 100 μL whole blood sample was mixed with 400 μL of acetonitrile, and 100 μL of the resulting mixture was transferred into the direct capillary spray reagent kit (Suzhou Purspec Technology, China). Each sample was analyzed in 10 replicates in the Cell portable MS, with a total detection run time of approximately 1.5 min per sample.

#### 2.5.4 Analytical methodology validation

The analytical method validation followed the guidelines of the International Council for Harmonisation of Technical Requirements for Pharmaceuticals for Human Use, assessing specificity, linear range, limit of detection (LOD), limit of quantification (LOQ), precision, accuracy and 1-h stability (14).

#### 2.5.5 PK-PD model establishment

PK-PD model analyses were performed using Phoenix WinNonlin software (Version 8.3.5, Certara, USA). The PK analysis utilized the compartmental modeling module. For C_b-Rocur_ below LOQ, values were set to 0 prior to reaching C_max_ and were considered unquantifiable after reaching C_max_. PD modeling used TOFR as the PD effect and was fitted using an inhibitory E_max_ model. If the coefficient of variation (CV) of estimated parameters was <20%, the precision of the estimated model parameters was deemed acceptable.

## 3 Sample size and statistical analysis

As a preliminary study, the sample size was limited to four beagles. Statistical analysis was performed using Graphpad Prism software (Version 9.5, Boston, USA). Data normality was assessed using the Shapiro–Wilk test. Measurement data were expressed as mean ± standard deviation (SD) or median (interquartile range, IQR). The standard curve was fitted using a linear regression equation based on data from three independent analytical batches. Within the linear range, the theoretical Rocur concentration was set as the independent variable (*x*), while the peak height of Rocur was the dependent variable (*y*). Weighted least squares regression was performed using a 1/*x* weighting factor. The fit goodness was evaluated using *R*^2^, where *R*^2^ ≥ 0.99 indicating a strong linear relationship. The statistical significance of the y-intercept was assessed via the 95% confidence interval (CI); if 0 fell within the 95% CI, the *y*-intercept was considered statistically non-significant. The regression equation was considered well-fitted if the relative standard deviation (RSD) of calculated concentrations relative to theoretical values was <20%. For consistency comparison, the Bland–Altman method was used to evaluate agreement. A scatter plot was constructed with the difference between the measured and predicted values on the *y*-axis and their mean on the x-axis, with the 95% CI indicated. Consistency was considered acceptable if the differences fell within the 95% CI, the mean difference line was close to 0, and bias was minimal. A *p*-value of <0.05 was considered statistically significant.

## 4 Results

### 4.1 Analytical methodology validation

#### 4.1.1 Specificity

**Figure 2:**
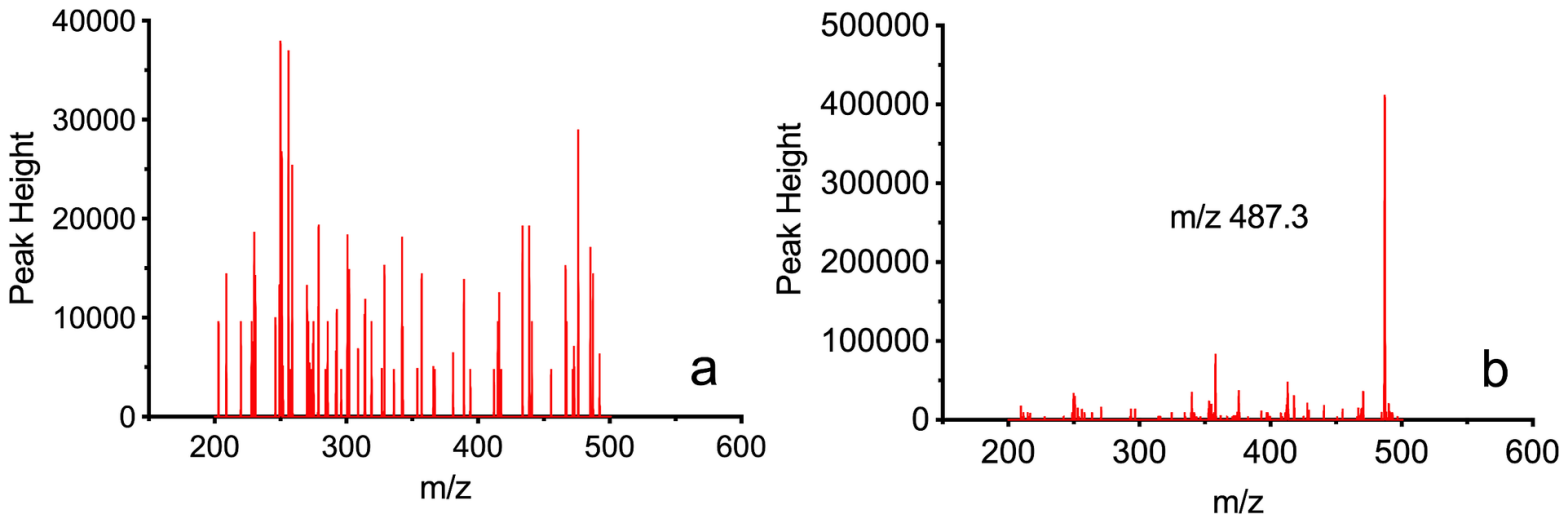
Full-scan mass spectrum of Rocur in blood samples measured using the Cell portable MS. **(a)** Depicts the full-scan mass spectrum of blank blood samples, whereas **(b)** presents the full-scan mass spectrum of blank blood samples mixed with Rocur. The fragment ion of Rocur was m/z 487.3. The results demonstrate that no significant interference was observed at Rocur’s detection site.

As shown in Figure 2a, presents the full-scan mass spectrum of blank blood samples, while Figure 2b shows the full-scan mass spectrum of blank blood samples mixed with Rocur. The results indicate no significant interference at the detection site for Rocur. Table 1 presents the validation parameters for the analytical method.

**Table 1:** Validation parameters for the analytical method.

Parameters	Results
Specificity	Full-scan spectra show no significant interference at m/z 487.3.
Linear range	50–10,000 ng/mL, *y* = 1108.32*x + 14873.99 (*R*^2^ = 0.993)
LOD	10 ng/mL at a signal-to-noise ratio of 3
LOQ	50 ng/mL at a signal-to-noise ratio of 10
Precision	RSDs were 8.85% at 7500 ng/mL, 9.40% at 5000 ng/mL, and 14.43% at 2500 ng/mL
Accuracy	Recovery rates were 92.16% at 7500 ng/mL, 87.26% at 5000 ng/mL, and 104.49% at 2500 ng/mL
1-h Stability at 25°C	Recovery rates were 97.86% at 5000 ng/mL, 95.43% at 500 ng/mL, and 88.66% at 100 ng/mL

LOD, limit of detection; LOQ, limit of quantification; RSD, relative standard deviation.

#### 4.1.2 Linear range

**Figure 3:**
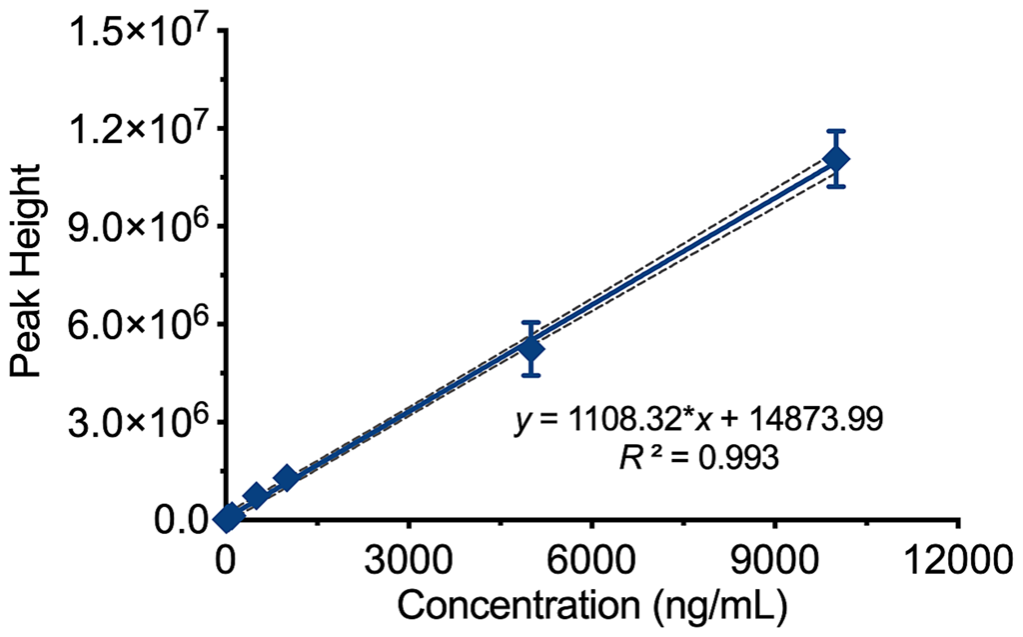
Linear range of Rocur measured using the Cell portable MS. The theoretical concentration (*x*) and peak height at m/z 487.3 (*y*) were analyzed using weighted least squares regression with a 1/*x* weighting factor. Rocur exhibited strong linearity within the 50–10,000 ng/mL range, described by the equation *y* = 1108.32*x* + 14873.99 (95% CI: −97,110 to 199,552), with *R*^2^ = 0.993.

Standard blood samples with concentrations of 10, 20, 50, 100, 500, 1,000, 5,000, and 10,000 ng/mL were prepared and processed according to the sample preparation procedure before being analyzed. As shown in Figure 3, the theoretical concentration was used as the independent variable (*x*), and the peak height at m/z 487.3 was used as the dependent variable (*y*). The weighted least squares regression was performed with a 1/*x* weighting factor. The results indicated a good linear relationship in the concentration range of 50–10,000 ng/mL for Rocur, described by the regression equation *y* = 1108.32*x + 14873.99 (95% CI = −97,110 to 199,552), with an *R*^2^ of 0.993. The RSD of the calculated concentrations compared to the labeled values ranged from 6.61 to 19.98%.

#### 4.1.3 LOD and LOQ

The average background noise value at m/z 487.3 in blank whole blood was measured as 5356.20. Based on a signal-to-noise ratio of 10, the LOQ was calculated as 50 ng/mL, and based on a signal-to-noise ratio of 3, the LOD was determined to be 10 ng/mL.

#### 4.1.4 Precision and accuracy

Three concentrations-high (7,500 ng/mL), medium (5,000 ng/mL), and low (1,000 ng/mL)-were selected, with three parallel samples prepared for analysis at each concentration to evaluate precision and accuracy. Precision was assessed using the RSD, while accuracy was evaluated based on the recovery rate. The results demonstrated that the method’s precision (RSD%) ranged from 8.85 to 14.43%, while the recovery rate was between 87.26 and 104.49%.

#### 4.1.5 1-hour stability

Simulated blood samples were prepared at concentrations of 5,000 ng/mL, 500 ng/mL and 100 ng/mL. After storage at 25°C for 1 h, the samples were processed and analyzed. The recoveries ranged from 88.66 to 97.86% of the labeled concentrations.

### 4.2 Consistency comparison

**Figure 4:**
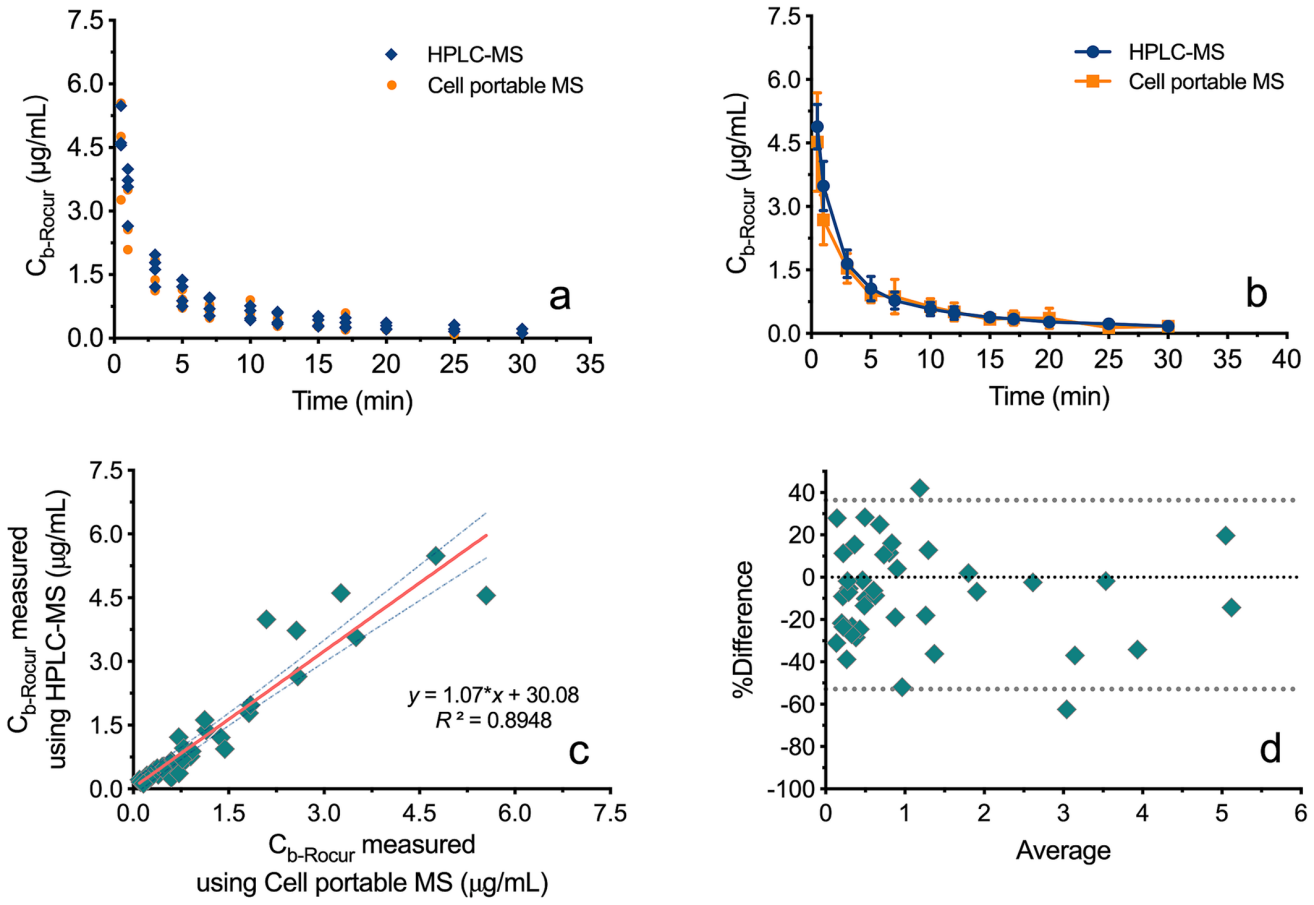
Comparison of Rocur concentrations measured by HPLC-MS and the Cell portable MS. **(a,b)** Illustrate the C_b-Rocur_-time curves measured by HPLC-MS and the Cell portable MS. **(c)** Presents a regression model incorporating all measured values, demonstrating a linear correlation (*R*^2^ = 0.8948). **(d)** Shows that data points in the Bland–Altman plot cluster near the mean difference line without a clear trend, with most points falling within the 95% CI.

This study included four beagles. Figures 4a,b both illustrates the C_b-Rocur_-time curve measured by HPLC-MS and the Cell portable MS. The C_b-Rocur_-time curves from both methods indicate that after a single injection of 3 ED₅₀ of Rocur until recovery, C_b-Rocur_ in all beagles increased rapidly and then declined. The C_max_ values were 4.52 ± 1.16 μg/mL (measured by the Cell portable MS) and 4.89 ± 0.52 μg/mL (measured by HPLC-MS), respectively. The mean concentration RSD for both methods was <15%. A regression model based on all measured values from both methods showed a linear correlation (*y* = 1.07*x + 30.08, *p* < 0.0001, *R*^2^ = 0.8948), as shown in Figure 4c. Figure 4d illustrates that the data points in the Bland–Altman plot are clustered near the mean difference line without any apparent trend, with most points falling within the 95% CI, indicating the absence of proportional bias.

### 4.3 PK-PD model establishment based on the cell portable MS

**Figure 5:**
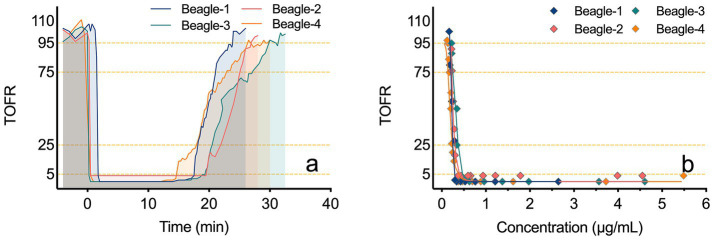
TOFR-time curves and PK-PD models of TOFR and C_b-Rocur_ measured by the Cell portable MS. **(a)** depicts the TOFR-time curves for four beagles after a bolus injection of Rocur. **(b)** presents the PK-PD model correlating C_b-Rocur_, as measured by the Cell portable MS, with TOFR values in four beagles.

Figure 5a illustrates the TOFR-time curves for four beagles following a bolus injection of Rocur. Figure 5b illustrates the PK-PD model between the C_b-Rocur_ determined by the Cell portable MS and the TOFR values for four beagles. Table 2 presents the parameters for the PK-PD model. The results indicate that after a single injection of 3 ED₅₀ of Rocur until recovery, no significant delay was observed compared with TOF. To account for this, a direct connection model was applied to the PK-PD model. The PK model followed a two-compartment model. Rocur rapidly distributed from plasma to tissues after administration, showing an initial rapid decline followed by a slower elimination phase. The half-life was approximately 10.88 ± 1.33 min. The volume of distribution in the central compartment was 1.45 ± 0.35 L/kg, while the peripheral compartment was 2.11 ± 0.75 L/kg. The clearance rates for the central and peripheral compartments were 0.37 ± 0.10 L/h/kg and 0.42 ± 0.13 L/h/kg, respectively. For the PD component, an inhibitory sigmoid E_max_ model was employed to accurately capture the trend of TOF responses. Under baseline conditions, the TOFR value was 102.47 ± 10.92. At peak blood concentration, the TOFR reached its lowest level, indicating profound muscle relaxation (TOFR <5%). As the C_b-Rocur_ decreased, the TOFR gradually recovered. The half-maximal muscle relaxation effect occurred at a C_b-Rocur_ of 0.25 ± 0.05 μg/mL. When the TOFR value first increased above 5% (within the 5–25% range), the C_b-Rocur_ was 0.30 ± 0.03 μg/mL; for the 25–75% range, the C_b-Rocur_ was 0.24 ± 0.03 μg/mL; for the 75–95% range, the C_b-Rocur_ was 0.21 ± 0.03 μg/mL; and for values exceeding 95%, the C_b-Rocur_ was 0.20 ± 0.03 μg/mL.

**Table 2:** Parameters for the PK-PD model.

Parameters	Results
PK model	Two-compartment model
PD model	Inhibitory Sigmoid E_max_ model
Link model	Direct connection model

	Estimated	CV, %
V_1_, L/kg	1.45 ± 0.35	2.63
V_2_, L/kg	2.11 ± 0.75	10.32
CL_1_, L/h/kg	0.37 ± 0.10	3.13
CL_2_, L/h/kg	0.42 ± 0.13	7.60
IC_50_, μg/mL	0.25 ± 0.05	7.27
γ	8.78 ± 2.01	17.69
E_0_, %	102.47 ± 10.92	6.85

Fitted value data are presented as the mean ± standard deviation. CV, coefficient of variation; V_1_, volume of distribution in the central compartment; V_2_, volume of distribution in the peripheral compartment; CL_1_, clearance rate for the central compartment; CL_2_, clearance rate for the peripheral compartment; IC_50_, half-maximal muscle relaxation effect concentration; γ, steepness; Ε_0_, baseline effect.

## 5 Discussion

Developing rapid detection methods is essential for facilitating individualized precision dosing and improving anesthesia safety. In clinical anesthesia, anesthesiologists typically base drug administration on the dose–response relationship rather than the concentration-effect relationship (15), primarily due to the absence of practical rapid drug concentration monitoring methods. Once intravenous anesthetics are administered, their concentrations in the bloodstream cannot be directly observed by anesthesiologists, unlike the minimum alveolar concentration (MAC) of inhaled anesthetics, which can be readily detected via the infrared sensor module in anesthesia monitors. Even with identical doses, interpatient variability in absorption, distribution, metabolism, and excretion results in significant differences in blood concentrations. Thus, underdosing or overdosing frequently occurs, leading to a high incidence of postoperative RNMB, with multicenter surveys (RECITE) reporting RNMB rates of 57.8 and 64.7% following extubation in general anesthesia surgeries (16, 17). Current detection methods mainly include LC (3), LC–MS (18), and gas chromatography–mass spectrometry (GC–MS) (19), offering high sensitivity and accuracy. However, their large size, high noise levels, labor-intensive and time-consuming sample pretreatment processes limit their application in operating rooms. In addition, drug-specific mobile phase systems and chromatographic columns add further complexity, making these methods unsuitable for anesthesiologists without specialized training (20).

Currently, studies on the rapid detection of Rocur are limited, with only a few reports focusing on electrochemical methods (8, 21). Cheng et al. designed N,N,N-trimethyl-4-(pyrrolidin-1-yl)butylammonium bromide (PyBTA) as a probe to detect Rocur through fluorescence intensity changes induced by displacement from sugammadex. This method demonstrated a linear range of 0.5–10 μM and a LOD of 0.3 μM. However, PyBTA’s fluorescence performance is susceptible to variations in experimental conditions such as pH and temperature, potentially affecting detection accuracy. Additionally, unknown interfering substances in real samples could alter fluorescence intensity, hindering precise quantification (22). Similarly, Fahem et al. developed a printed potentiometric sensor incorporating a calix[6]arene ion carrier, which showed a linear response to Rocur concentrations ranging from 1 μM to 10 mM with high selectivity. Nevertheless, this method requires intricate manufacturing techniques and specialized technical support, limiting its practical clinical adoption. Moreover, although the sensor is highly selective for Rocur, it may still be influenced by unknown interferences in complex biological samples. Its long-term stability and performance across diverse clinical environments require further validation (21). In this study, we utilized the Cell portable MS, which employs a linear ion trap mass spectrometer. Its compact size (measuring 333 × 235 × 146 mm and weighing <8.5 kg), stable performance, and suitability for use in space-constrained environments such as operating rooms make it ideal for long-term clinical application.

In addition to its highly compact design, the Cell portable MS eliminates the need for complex sample pretreatment. Our preliminary research explored the possibility of monitoring Rocur in exhaled breath, similar to propofol or ciprofol. However, no evidence was found to indicate that Rocur is excreted via exhalation, as neither exhaled breath nor condensate contained detectable drug levels. Therefore, blood remain the primary medium for determining its drug levels. Blood samples, however, often contain impurities such as salts and matrix components, significantly compromising the sensitivity and quantitative accuracy of MS detection. As a result, time-consuming and intricate pretreatment steps are usually required (23). Recently developed techniques, such as direct sampling with metal probes, enable the adsorption of analytes. However, the amount of adsorbed analyte is often insufficient for detecting low-concentration targets, and solid materials carried by the probe can clog the sample inlet, further complicating the analysis (24). Similarly, solid-phase microextraction methods are time-intensive, have low extraction recovery rates, and exhibit selectivity, which may lead to the loss of critical chemical information (25). The Cell portable MS overcomes these challenges by utilizing an in-situ ionization reagent kit. The kit’s sample substrate adsorbs impurities, such as proteins, from blood samples, effectively separating and purifying the target drug.

The linear range established in this study, 50–10,000 ng/mL, encompasses the clinically reported concentration range for Rocur (26). The RSD for concentrations ranged <15%, meeting the requirements for biological sample analysis (27). The analysis results (*R*^2^ = 0.8948) of the Cell portable MS were highly consistent with those of HPLC-MS, demonstrating the high accuracy of the Cell portable MS. This capability enables timely monitoring of NMB and the detection of residual effects. Moreover, this trace blood sampling method, compared to electrical stimulation, is better suited for awake patients. Given the application scenario of rapid post-sampling detection, we conducted a 1-h stability analysis of the samples. The results indicated that the samples were relatively stable within this timeframe. It should be noted that the method used in this study was based on external standard calibration. Although verapamil has been reported as an internal standard in previous studies (28), our preliminary trials revealed inconsistent matrix effects under this method. However, since the standard curve was constructed using the same blood matrix and parameters, its results can be directly applied to the analysis of final blood samples. The absence of an internal standard is a limitation of this bioanalytical method. Future studies should explore alternative internal standards to minimize variability from factors such as instrument stability and analytical conditions.

In this study, we observed that the duration of NMB was relatively prolonged. On one hand, to simulate clinical dosing and achieve complete NMB after a single administration, the injected dose was set at 3 times the ED_50_ (29). On the other hand, the prolonged blockade might also be associated with the use of propofol. Chen et al. reported that the average duration of NMB induced by Rocur was extended when combined with propofol at 1.25 times the minimum infusion rate (30). From a PK perspective, the sampling points did not encompass the full elimination phase following recovery from NMB, leading to the PK model of Rocur conforming to a two-compartment model. Despite the inclusion of only four experimental animals in this study, the parameters showed good consistency across subjects.

Research has shown that NMB induced by Rocur does not affect the patient state index in anesthetized dogs (31). Consequently, conventional vital sign monitoring is inadequate for accurately assessing the degree of NMB, highlighting the need for specific monitoring during anesthesia. However, AMG has notable limitations, including reduced measurement precision, frequent calibration requirements, time-intensive procedures, susceptibility to external interference, and reliance on specific muscle groups. Additionally, the high stimulation intensity reduces acceptability in conscious patients. Studies have also reported that RNMB (TOFR values between 0.7 and 0.9) can lead to postoperative hypoxemia and pulmonary complications (32). To accurately assess the neuromuscular effects of Rocur, we controlled interference factors in this study. The results demonstrated good modeling performance and high data consistency between different blood concentrations and TOF effects across profound blockade, deep blockade, moderate (surgical) blockade, and recovery stages. Furthermore, the PD model we developed suggests that C_b-Rocur_ measured by the Cell portable MS correlate strongly with TOF effects. This relationship was evident as TOFR was concentration-dependent. Previous studies have quantitatively measured unbound Rocur in muscle tissue, demonstrating that parameterized PK-PD models can reliably estimate effect-site concentrations (33). Our findings indicate that rapid blood concentration detection facilitates individualized drug concentration monitoring and modeling, aiding in precise dose adjustments.

This study has several limitations. First, although the Cell portable MS provides a preliminary approach for the rapid detection of Rocur and the development of PK-PD models, the small sample size and intensive sampling strategy may introduce variability, such as sampling and analytical errors, potentially affecting the robustness and generalizability of the PK-PD model. Future studies could adopt population-based sampling strategies with a larger cohort to improve model reliability and applicability. Second, the risk of RNMB is significantly influenced by patient-specific factors, including age and the duration of Rocur administration. Age-related physiological changes can alter drug metabolism, with elderly patients often experiencing prolonged NMB due to impaired clearance. Additionally, prolonged neuromuscular blocker administration can lead to drug accumulation, further increasing the risk of RNMB. Given the inherent limitations of animal models, they may not fully replicate human metabolic pathways or the pharmacodynamic responses observed in clinical practice. Therefore, well-designed clinical studies are essential for validating these findings and ensuring their applicability to real-world anesthesia practice. Third, clinical anesthesia typically involves multiple agents, which may exert synergistic or antagonistic interactions, thereby influencing the pharmacokinetics and pharmacodynamics of administered drugs. In this study, propofol was used to simulate clinical anesthesia; however, other commonly used adjunctive agents, such as opioids, benzodiazepines, and additional muscle relaxants, were not included, despite their significant impact on the pharmacodynamics of Rocur. The absence of these agents limits the generalizability of our PK-PD models to actual clinical anesthesia settings. Future studies should consider the interactions among various anesthesia-related drugs to better characterize rocuronium’s behavior to enhance the predictive accuracy of PK-PD models. Lastly, the Cell portable MS relies on discrete-point detection, lacking the capability for real-time monitoring. Future advancements in sample injection methods are needed to enable automated and rapid analysis, improving the system’s applicability for real-time drug monitoring in clinical practice.

## 6 Conclusion

This study successfully applied the Cell portable MS for rapid quantitative analysis of Rocur concentrations in whole blood, demonstrating good consistency with the traditional HPLC-MS method. The findings revealed the good correlation between the PK-PD properties of Rocur and TOF effects, providing quantitative support for optimizing clinical drug administration strategies and potentially reducing the risk of postoperative RNMB.

## Data Availability

The original contributions presented in the study are included in the article/supplementary material, further inquiries can be directed to the corresponding author.
